# Dynamic complex opto-magnetic holography

**DOI:** 10.1038/s41467-022-35023-9

**Published:** 2022-11-26

**Authors:** M. Makowski, J. Bomba, A. Frej, M. Kolodziejczyk, M. Sypek, T. Shimobaba, T. Ito, A. Kirilyuk, A. Stupakiewicz

**Affiliations:** 1 Faculty of Physics, Warsaw University of Technology, 75 Koszykowa, 00-662 Warsaw, Poland; 2 Faculty of Physics, University of Bialystok, 1L Ciolkowskiego, 15-245 Bialystok, Poland; 3 Graduate School of Engineering, Chiba University, 1-33 Yayoi, Inage, Chiba, 263-8522 Japan; 4 FELIX Laboratory, Radboud University, 7 Toernooiveld, 6525 ED Nijmegen, The Netherlands

**Keywords:** Optical techniques, Ultrafast photonics, Displays

## Abstract

Despite recent significant progress in real-time, large-area computer-generated holography, its memory requirements and computational loads will be hard to tackle for several decades to come with the current paradigm based on a priori calculations and bit-plane writing to a spatial light modulator. Here we experimentally demonstrate a holistic approach to serial computation and repeatable writing of computer-generated dynamic holograms without Fourier transform, using minimal amounts of computer memory. We use the ultrafast opto-magnetic recording of holographic patterns in a ferrimagnetic film with femtosecond laser pulses, driven by the on-the-fly hardware computation of a single holographic point. The intensity-threshold nature of the magnetic medium allows sub-diffraction-limited, point-by-point toggling of arbitrarily localized magnetic spots on the sample, according to the proposed circular detour-phase encoding, providing complex modulation and symmetrical suppression of upper diffractive orders and conjugated terms in holographically reconstructed 3-D images.

## Abstract (web summary)

Holography recreates both the amplitude and wave front of a three dimensional object, meaning that the observer perceives the image in the nearly same way as they would the true object. Creating such holographic images is challenging computationally, and requires extremely fast display update. Here, the authors combine a fast memoryless computation algorithm with the ultra-rapid writing based on all-optical switching of a ferrimagnetic film.

## Introduction

Computer-generated holograms (CGH) with their animated, three-dimensional appearance have long appealed to our imagination as the path towards truly immersive displays with bi-directional, natural parallax. Impressive progress in updateable 3-D imagery^1^ has been achieved with liquid crystal modulators^2^ and high-resolution, but quasistatic holograms are being recorded in photosensitive materials^3–5^. Ultimate large-scale holographic displays based on CGH will require an exotic light modulation device, capable of rewriting as many as 10^12^ light-diffracting cells^6^ at a minimal framerate of 180 Hz, assuming color field sequential operation. To date, no large-area material or method having such properties has been demonstrated. Liquid crystal on silicon^7^ spatial light modulators^8^ (LCoS SLM) offers short refresh times in the order of microseconds in the case of ferroelectric LC^9^. However, they have limited sizes in the order of centimeters, and moreover their Spatio-temporal product^6^ is restricted by the fundamental limitations of LC and ineffective parallel data transfer scheme of pixel-driving circuitry with time-consuming row-column serial addressing. Using multiple SLMs in both coherent^10^ and incoherent matrices^11^ partly overcomes this trade-off by offering, e.g., 133 megapixels from 16 SLMs^11^, but with a penalty of increased complexity^12,13^ and image inconsistency. Photorefractive and photochromic materials^4,14^ allow large-surface, ultra-dense, pixel-less writing. However, their long response times in milliseconds and relatively long nanosecond pump pulses with fluences^3^ of 650 mJ cm^−2^ effectively render them quasistatic. From the standpoint of scalability, the ability to achieve dense writing on large areas without any in situ electrodes is essential. This is feasible with two interfering beams^15^ at µs speeds. However, in addition to the bulky optical setup and high voltages involved^16^, this does not ensure angular flexibility in the positioning of writing areas, unless mechanical sample movements are involved^3^. Moreover, owing to the sine profile of the recorded fringes, stray orders of diffraction are formed in the replay images. In contrast, single-beam point-by-point writing has greater flexibility and scalability. Nevertheless, micrometer positioning and localization of spots on a sample typically requires extremely large numerical apertures^17^. Apart from the optical and material challenges, computing a CGH as a whole in the form of a large matrix of complex numbers is inevitably constrained by the available random-access memory (RAM) and by limited capabilities to compute two-dimensional fast fourier transforms, despite recent progress^18–20^. Although numerous real-time solutions have been presented to date, the transition towards billions and eventually trillions of CGH points requires a change of paradigm in CGH computation, storage, and rewriting.

In this work, we propose to serially compute and instantly write a single CGH point at a nanosecond time scale with a dedicated hardware FPGA (Field Programmable Gate Array) unit fed with data describing the spatial coordinates and intensities of the cloud-point representation of the input 3-D scene^21^.

## Results

### Computation and opto-magnetic writing of CGH

**Fig. 1:**
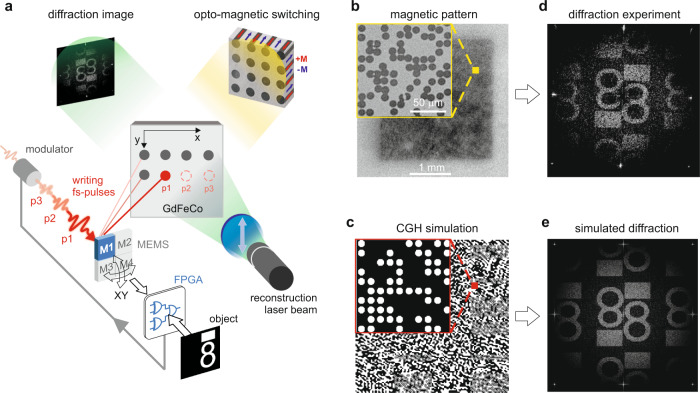
Serial writing of a CGH in an opto-magnetic medium. **a** Point-by-point optical switching of magnetic spots in GdFeCo sample with subsequent pulses *p*1-*p*3 from a femtosecond laser with a fluence of <20 mJ cm^−2^, reflected by a scanning MEMS mirror (M1), running free along raster-like or resonant (Lissajou) paths. M1 reports its current angles to the FPGA unit, which computes on-the-fly the binary triggering signal for the intensity modulator of the femtosecond laser beam; the FPGA unit is fed with cloud-point representation of the input object, constraint to *N* = 2048 object points in this implementation due to limited number of gates in the FPGA chip; geometrical imaging of the sample with a white polarized light using magneto-optical Faraday geometry; far-field diffraction image observed on a camera after passing of the 532 nm reconstruction laser beam through the sample, an analyzer, and a focusing lens. **b**, **c** Magnetic pattern written and simulated in the sample comprising 256 × 256 CGH points; **d**, **e** experimental and simulated far-field diffraction images showing the reconstructed image of the input object accompanied by twin conjugate image due to binary amplitude modulation. The diffractive efficiency is estimated at 5%. Partial suppression of upper order images is observed due to minor randomization of positions of CGH points in the sample^53^. The circular spots allow symmetrical and homogeneous suppression of higher-order diffractive terms in far-field, as opposed to square CGH pixels (see Methods), which could be enforced e.g. by beam shaping of the writing fs laser.

The serial single-point computation is done in two stages (see Methods). First, the phase contributions of all point sources in the input scene are computed in parallel by separate circuits in the FPGA module in relation to the current CGH cell to be written, which is targeted by current angles of the micro-electro-mechanical (MEMS) mirror scanning the writing laser beam over the hologram (see Fig. 1a). Subsequently, the phase contributions are counted by the *popcount* unit and the final binary value is delivered, triggering a single 35 fs pulse of the writing laser. Thus, instead of being saved in RAM, the computed CGH point is immediately written in the opto-magnetic GdFeCo film (see Methods) in the form of a locally reversible, all-optically switched (AOS) magnetic spot, as shown in Fig. 1. Hence, each CGH point, being a derivative of contributions from all object points, is computed within one cycle of the FPGA clock. Based on the given set of input object points any arbitrary number of CGH points can be calculated and written. The mechanism of ultrafast AOS, independent of the laser polarization^22^ in a large spectral range, allows a stable magnetic spot to be written and rewritten after an interval of only 30 ps^23^. This process, which is equivalent to storing data in non-volatile memory at a picosecond time scale, enables ultrafast writing of subsequent CGH points in other arbitrary positions on the sample. This feature differentiates this unique material from other non-volatile media, such as photopolymers that typically require exposure times greater than nine orders of magnitude^24^.

Magnetic spot switching in the GdFeCo sample occurs above a certain intensity-threshold with size-dependent on the intensity of the femtosecond laser pulse. Because a symmetric Gaussian beam is used for writing, well-defined circular areas are switched (see Fig. 1a) with diameters falling below the diffraction limit^23,25^. This feature allows the dense packing of highly localized CGH spots. Reversible point-by-point writing of holographic patterns by phase change of Ge_2_Sb_2_Te_5_ was reported^26^ with a numerical aperture (NA) of 0.8. In contrast, the proposed opto-magnetic method uses low NA of 0.01 that is supported by the said threshold effect, allowing convenient recording of CGH spots with sizes below *d* = 10 µm at a working distance of 100 mm (see Methods). The final size of the hologram will be limited by the product of the working distance and achievable angles of the MEMS. The density of CGH writing in the demonstrated part of the sample was 100 points per mm (see Fig. 1b), which could be estimated to ~2400 × 2400 points (24 × 24 mm^2^) for full available angular range of the MEMS (±5°). From here our method can be easily scaled up in the hologram size and its writing density. The former can be done by a MEMS with a larger scanning range, by setting up multiple MEMS mirrors in parallel (each illuminated with a separate laser beam), by increasing the working distance, or by any combination of the above. Notably, while extending the angular scanning range the low NA conveniently helps with the beam focusing due to the natively large depth of focus. Separately, the density of writing can be scaled by decreasing the spot sizes by deliberately lowering the intensity of the writing laser pulses, combined with more frequent pulsing while the MEMS scans over the sample. The limitation of this approach is the precision of the intensity modulator, which translates to more notable errors in spot sizes due to strong utilization of the threshold effect in the material. In time domain, the speed of femtosecond laser beam scanning can considerably exceed the inertial limits of a given MEMS^27^; therefore, the pace of holographic recording is limited only by the repetition rate of the laser and the clock of the FPGA (availability up to 1 GHz), whichever is slower. Although a laser with 1 kHz repetition was used in this initial demonstration, a gigahertz femtosecond laser potentially allows non-volatile rewriting of CGHs comprising 5.6 million points at 180 Hz framerate with the proposed method. This would be superior to typical Full-HD LCoS SLMs that feature a similar framerate but having only 1920 × 1080 pixels (2.1 million).

### Rewriting of holograms

**Fig. 2:**
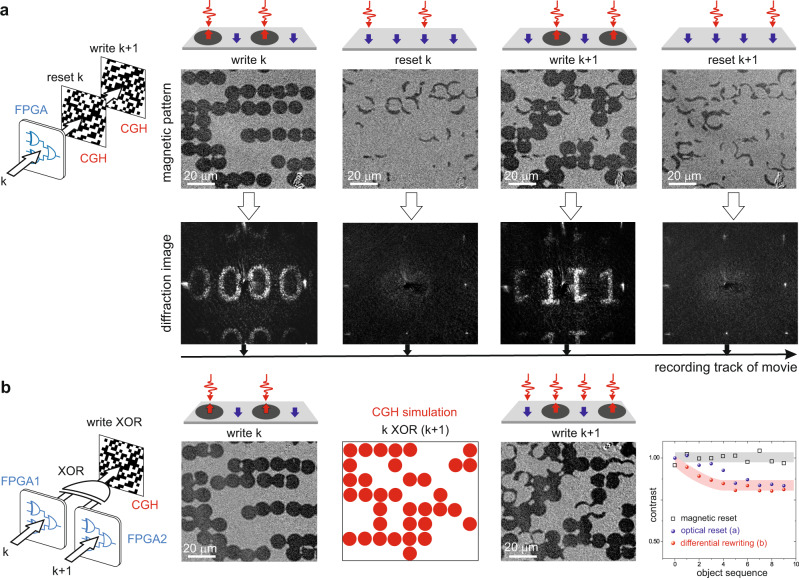
Dynamic opto-magnetic rewriting of subsequent holographic frames. **a** Optical reset mode comprising subsequent writing of *k* and *k* + 1 holographic patterns using toggle regime of AOS. From left to right: writing of pattern *k* required 40 exposures (fragment of the real magnetic pattern is shown); reset stage toggled the magnetic spots to their initial state with extra 40 exposures; writing of next pattern *k* + 1 required 34 exposures; second reset stage as a part of track of movie required another 34 exposures (148 exposures in total for rewriting). **b** Differential rewriting mode. From left to right: computation scheme using two separate FPGA units; writing of *k* pattern required 40 exposures; computation of the logical XOR between *k* and *k* + 1 frames; writing of *k* + 1 frame required 35 exposures (the differential mode allowed an almost twofold, i.e., 148/(35 + 40) reduction of the number of required exposures); experimental contrast of reconstructed images (see Methods) as a function of rewriting sequences for all rewriting modes. The middle panel of diffraction images shows a movie of holographic frames (see Supplementary Movie 1). The visible remanent magnetic domains in images of the magnetic pattern after resetting are caused by long-lived motions of domain walls within milliseconds after AOS. This magnetostatic effect causes minor off-axis background noise and in consequence, the reduction of the contrast of holographic playback frames becomes negligible after a sequence of four write-reset acts, as shown in the graph. The colored bands in the graph represent the moving average value and their thickness is equal to the standard deviation doubled. The size of the shown magnetic patterns is 106 × 96 µm^2^.

Dynamic updating of optically written holographic frames can operate in three modes: (i) globally using a coil-induced external magnetic field; (ii) by all-optical serial restoring of all magnetic spots to the initial state (see Fig. 2a); and (iii) selectively by differential exposures (see Fig. 2b). Although all modes exhibit similar performance (see inset in Fig. 2b), here we focus on the optical modes, having the practical benefits of operating remotely and without any external fields. The third mode comprises all-optical toggling^28^ of the magnetic states limited to those CGH points selected by the logical XOR operation between *k* and *k* + 1 states of the input 3-D scene. Although this approach doubles the number of required FPGA units, it results in an approximately twofold reduction in the number of femtosecond laser exposures, allowing the refreshing of a CGH at the same framerate with twice as many points. As a manifestation of the unique holographic feature, the complete images are reconstructed in far-field diffractive plane while the point-by-point recording is still in progress, i.e., without strict division to subsequent holographic frames in time domain (see Methods). The opto-magnetic CGH recording throughput can be scaled up even further, without considerable limitations. This can be achieved by writing several areas of the sample simultaneously after adding more MEMS mirrors^13^ (marked as M1-M4 in Fig. 1a), each with a dedicated FPGA computation unit and a writing laser beam. As an example, using four MEMS scanners separately illuminated by a GHz laser, with double FPGA units per each scanner, could allow the rewriting of CGHs comprising ~4·2·5.6 = 44.8 million points at 180 Hz, thus enabling color-sequential operation with unprecedented resolutions. In such a case, operating at low NA values greatly facilitates the focusing of multiple writing beams on the hologram. The picosecond switching cycles of holographic cells have the potential to be used as ultra-rapid reconfigurable transmissive diffractive optical elements. They could replace ferroelectric LCoS SLMs or Digital Micromirror Devices in selected cases of optical trapping^29^, manipulation of optical vortices^30^, orbital angular momentum^31,32^, optical interconnections, multiplexers and switches^33^, reconfigurable add-drop multiplexers^34^ and electronic circuits^35^. For such purposes, useful sophisticated optical functionalities are achievable with CGHs containing as few as 64 × 64 points that can be potentially computed and updated at 2 MHz rates with four parallel MEMS-FPGA units fed by a GHz laser in future implementations.

### Circular detour-phase encoding

**Fig. 3:**
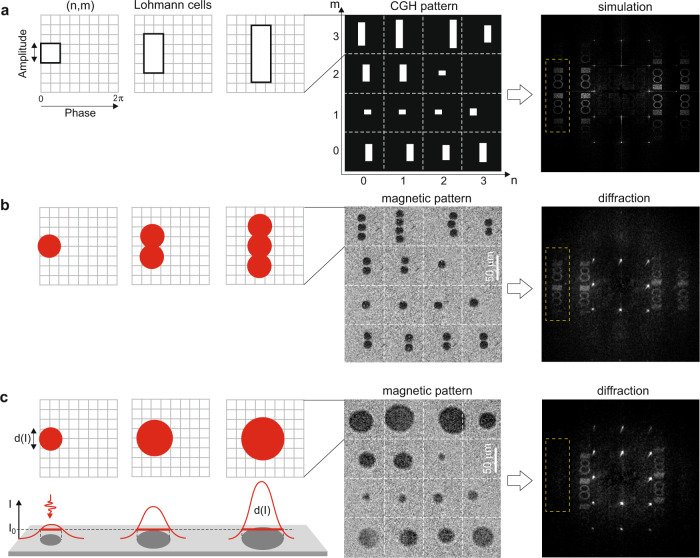
Complex opto-magnetic holograms with detour-phase encoding. **a** Simulation of classical detour-phase encoding using the Lohmann method. The heights and offsets of rectangular apertures are linear functions of the amplitude and phase values from a given CGH cell, respectively. Suppression of the conjugated images is notable. **b** From left to right: the approximation of the rectangular Lohmann openings with multiple point-by-point exposures switching magnetic spots of fixed diameters; fragment of written magnetic pattern; experimentally acquired diffraction image showing asymmetrical visibility of higher-order image duplicates (marked by a dashed yellow rectangle) originating from regularly spaced Lohmann cells. **c** From left to right: visualization of the writing of three adjacent Lohmann cells with circular switched magnetic spots of diameters set by in-the-loop modulation of the pump beam intensity (see Fig. 1); fragment of written magnetic pattern; experimental reconstruction showing the symmetrical suppression of higher-order image duplicates. The diffractive efficiency is estimated at 1%. The size of magnetic pattern images is 320 × 320 µm^2^.

The drawback of the opto-magnetic medium is the binary modulation of the playback light that inevitably induces conjugate images in the diffraction field^36^, as shown in Fig. 1. By introducing the Lohmann encoding^37^ we effectively switched to complex (amplitude and phase) modulation while still operating in binary amplitude medium. The non-pixelated nature and intensity-threshold AOS in the GdFeCo sample allows one to write in precise locations circularly shaped magnetic spots to form openings of Lohmann cells (see Fig. 3a). In the first attempt, we used the multiple AOS with constant diameters of magnetic spots to build approximations of the classical rectangular openings (see Fig. 3b). The experimental results demonstrate the elimination of conjugate terms and consistency with simulation, nevertheless the asymmetrical suppression of higher diffraction orders was also observed. In order to overcome this, we proposed the modified detour-phase encoding using single-shot circular openings (see Methods), which conveniently resulted in the symmetrical suppression^38^ of higher-order image duplicates in the far-field, as shown in Fig. 3c. The diameter of each opening *d*(*I*) was adjusted by modulating the intensity of the pump beam on-the-fly, which differentiates this approach from previous serial writing attempts with fixed spot sizes^26^. In addition, this feature reduces the total number of exposures per holographic frame up to three times, as compared to asymmetric detour-phase encoding seen in Fig. 3b.

## Discussion

The presented method operates at room temperature in non-contact mode using ultrashort laser pulses, which is greatly supported by the availability of all-fiber femto- and picosecond lasers operating within 1–10 GHz. It outperforms existing alternative approaches, enabling the fastest ever serial writing and optical refreshing of CGH in non-pixelated, large-area, easily manufacturable, transparent (or reflective) medium as an updateable holographic non-volatile memory. To our best knowledge, this is the first technique in which the memory requirements, computational complexity, and size of all used optical components do not scale up with the size and resolution of the hologram. Recent progress in material engineering that enables all-optical switching with longer picosecond pulses of low fluence, such as in dielectrics^39^ or metallic [Tb/Co] multilayers^40^ introduces a plethora of new possibilities. Furthermore, employing the proposed method in nanostructured media could be useful for volume multi-color holographic recording approaching sub-wavelength resolution^41^. Although CGH spot sizes in the range of 10 µm are presented here, denser writing in the sub-µm regime, ideal for holographic displays, could be achieved down to 60 nm with nano-engineered samples of similar composition^42^. The complexity of the cloud-point representation of the input object can be improved 40-fold with the existing hardware by the combination of FPGA-based recurrence algorithm and e-ASIC implementation (see Methods). These advantages combined with the scalable computation scheme and ultra-low requirements for computer memory open the way towards high-resolution holographic 3-D TV displays and ultra-wide angle near-eye augmented/virtual reality goggles^43^.

## Methods

### Materials

The measurements were performed on ferrimagnetic alloy with the composition AlTi(10 nm)/Si_3_N_4_(5 nm)/Gd_24_Fe_66.5_Co_9.5_(20 nm)/Si_3_N_4_(60 nm), prepared by magnetron sputtering on a glass substrate. The sample was 15 × 15 mm^2^ in size and optically transparent in the visible spectral range. The used alloy has perpendicular orientation of the magnetization and shows a square shape of the hysteresis loop with a coercive field of 75 Oe. Such alloy was used before for AOS with a single femtosecond laser pulse by toggle regime^22^. The AOS effect in GdFeCo alloys is driven by ultrafast and efficient thermal demagnetization at the characteristic time of about 30 ps after a single pump pulse. The mechanism of AOS in this type of samples^23^ provides a unique way of CGH recording as magnetic patterns with a spatial redistribution. We note that the magnetization switching does not depend on the pump polarization within the whole VIS-NIR spectral range.

### Recording of CGH magnetic patterns with femtosecond laser pulses

**Fig. 4:**
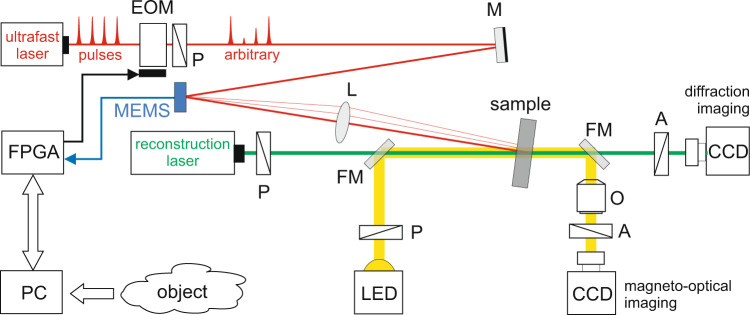
Scheme of the experimental setup. Outline of CGH writing and acquisition of diffraction and magneto-optical images using Faraday effect in a transmission geometry. L, convex lenses with a 100 mm focal length, FM flip mirror, P polarizer, A analyzer, O objective lens, M reflective mirror, CCD cameras, LED white light illumination, EOM electro-optical modulator.

We write and erase the magnetic patterns in the optically transparent GdFeCo film on a point-by-point basis with the use of MEMS (Mirrorcle A7M20) steered single linearly-polarized ultrafast pump pulses (see Fig. 4) with a duration of 35 fs, the central wavelength of λ = 800 nm and a maximum repetition rate of 1 kHz (Astrella, Coherent). In principle, the repetition rate of the laser pulses can be scaled up to 10 GHz frequency, which is limited only by the mechanism of AOS in GdFeCo alloy. The pump beam with the fluence below 20 mJ cm^−2^ was focused to a spot of 50 μm in diameter using a lens of *f* = 100 mm placed before the sample. The diameter of the pump beam at the lens was 2 mm, which is equivalent to the numerical aperture of 0.01. By lowering the pump beam intensity and taking advantage of the threshold effect, the optically switched areas of c.a. *d*_*s*_ = 10 μm were achieved in the same optical configuration, which was equivalent to NA = 1.22*λ*/(2*d*_*s*_) = 0.05. Further optimization of the sample composition and reduction of the size of spots will potentially allow writing with energy-efficient ultrafast all-fiber lasers^44^.

**Fig. 5:**
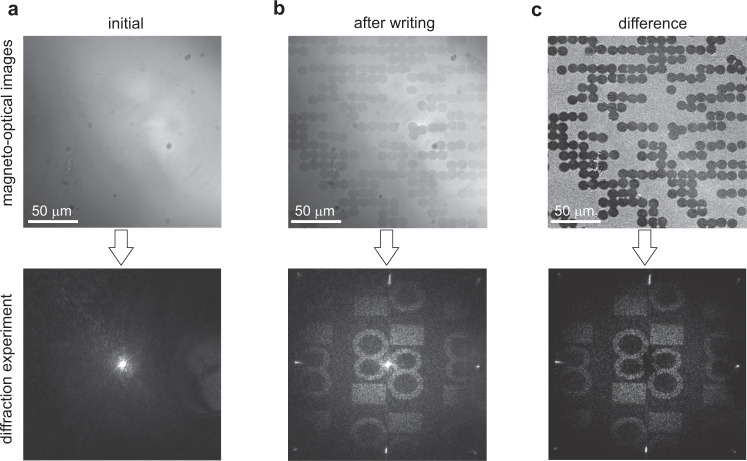
Opto-magnetic CGH writing and reading. From left to right shows both the magneto-optical (top panel) and diffraction (bottom panel) images in the GdFeCo sample **a** before the opto-magnetic writing, **b** after writing, and **c** the difference between these images. The magneto-optical images show an area of 200 × 200 µm^2^.

The magnetic pattern in the sample was visualized using a standard magneto-optical polarizing microscope. The polarized light source was a LED lamp with output routed into the optical path of the probe beam. The LED light then passed the sample and was gathered with an objective before passing an analyzer and hitting the CCD camera. The magnetic contrast in such a polarizing microscope comes from the fact that magnetic spots with different perpendicular magnetization orientations to the sample plane will give different rotation of the polarization plane (effect of Faraday rotation), and thus the light passing through them will acquire different polarization, which can be easily detected on the CCD camera. After recording, the pattern stays unchanged for a long time due to the non-zero coercivity in the medium. All measurements were done without applying external magnetic fields and at room temperature. The images of magnetic pattern and diffraction were taken before and after the CGH recording. We used the difference (standard procedure of magnetic image processing) of these images to visualize pure magnetic pattern in the magneto-optical images and subtract the zero-order light in the diffraction images (see Fig. 5). The initial background image before CGH recording was obtained after the application of a brief external perpendicular magnetic field with >80 Oe. Such magnetic field was also used to erase the entire recorded CGH magnetic pattern. The diameters of magnetic spots were adjusted by changing the pump intensity with an electro-optical modulator (EOM), assisted by a polarizer (see Fig. 4).

**Fig. 6:**
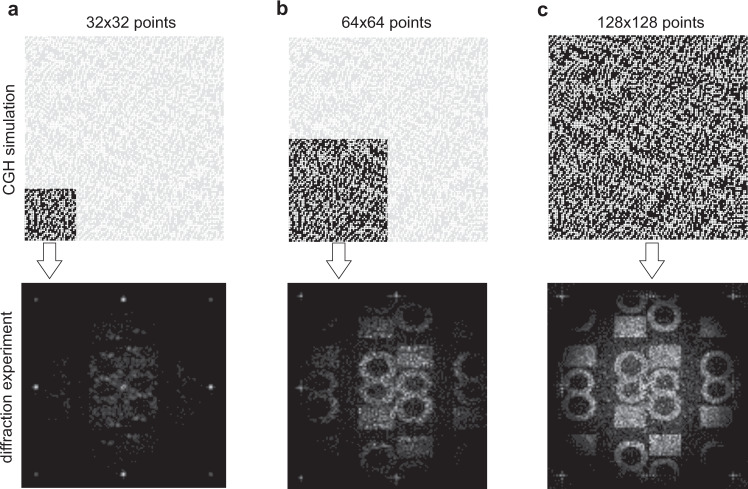
Progressing writing of holographic points. CGH pattern (top panel) and reconstructed far-field diffraction images during progressing writing of **a** 32 × 32, **b** 64 × 64, **c** 128 × 128 holographic points.

The sequence of CGH recording is simple. The current angles of the free-running MEMS mirror are reported to the FPGA unit. Based on the returned binary signal, a single laser pulse is transmitted through the intensity modulator (EOM) and switches the magnetization in the medium. The procedure is repeated immediately after the next angles of the MEMS are available, allowing uninterrupted point-by-point recording (see Fig. 1). Another laser pulse directed at the same written magnetic spot reverses it, allowing for a rapid change of the pattern into another one, with the possibility of reusing unmodified points. The hologram was recorded as domains with magnetization orientations opposite to the initial state, using laser-induced switching. The image encoded into the hologram was reconstructed in real-time during the writing process by means of the magneto-optical Faraday effect in the optically transparent sample (see Fig. 4). The magnetic sample has a large Faraday rotation angle, which results in a clearly recognizable diffraction images^45^. We note that during increasing the CHG writing density due to the Gaussian shape of the laser pulse the overlapping effect of magnetic spots was observed^46^. In this case, there was observed the toggle effect in areas between subsequent written spots. Despite this, the quality of far-field reconstructions of the written holographic patterns was unchanged. The overlapping effect could be useful for holographic writing of a practically unlimited number of CGH points with arbitrary fill factor values. We also note that the serial mechanism of CGH writing allows the appearance of the recognizable holographic image during unfinished rewriting of the holographic frame. In Fig. 6, we demonstrated the time trace of the displays diffraction image which is recognized for the minimal number of 32 × 32 points. This feature is observed only during serial writing and can be attributed to the non-volatile memory effect in the medium.

### Circular detour-phase encoding

**Fig. 7:**
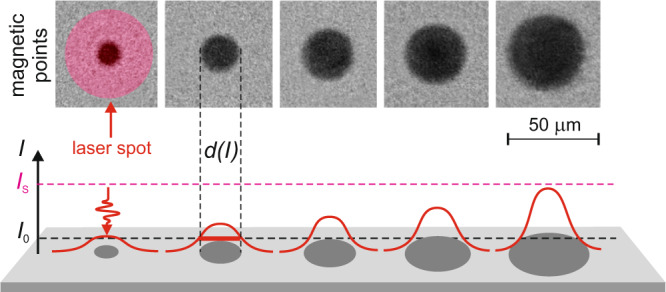
Writing single magnetic points using AOS. The images of reversible writing by AOS magnetic points with a different pump fluence from left to right show: 12 mJ cm^−2^; 14.2 mJ cm^−2^; 15.6 mJ cm^−2^; 17 mJ cm^−2^ and 20 mJ cm^−2^. The horizontal dashed lines indicate the switching threshold and saturation of the laser pulse intensity *I*. The laser spot also denotes the diffraction limit of the optical configuration of writing process.

The test holograms for complex encoding inspired by Lohmann method^37^ were computed as Fourier transforms of the input images having 512 × 512 pixels, with a random initial phase. For each of the 512 × 512 assumed Lohmann cells the amplitude and phase values were extracted, denoted as *a*(*n*,*m*) and *φ*(*n*,*m*), respectively. The writing beam was then positioned inside a given cell, offset from its center by the distance:

1 $$\delta x\left(n,m\right)=\varphi \left(n,m\right)\frac{\triangle x}{2\pi },$$

where Δ*x* = Δ*y* were the dimensions of each Lohmann cell. The diameter of the optically switched area *d*(n,m) was directly connected with the amplitude *a*(n,m) with the simple relation:

2 $$d\left(n,m\right)=\frac{b{{{{{\rm{|}}}}}}a(n,m){{{{{\rm{|}}}}}}}{\triangle x},$$

where the *b* = 0.7 margin was found in numerical optimizations in order to allow minimally invasive overlapping of Lohmann openings in adjacent cells^46^. The femtosecond writing beam had the gaussian intensity profile with waist diameter being a function of the used numerical aperture of the focusing optics. The appropriate intensity *I* of the writing beam was adjusted to induce the magnetically switched circular area of the desired diameter *d* (see Fig. 7), by matching *d*(*I*) = *d*(*n*,*m*) in a look-up table filled according to the below formula:

3 $$d\left(I\right)=2\sqrt{-\frac{1}{2}{w}_{S}{{{{{\rm{ln}}}}}}\left(\frac{{I}_{S}-I}{{I}_{S}-{I}_{0}}\right)},$$

where the minimal (threshold) intensity of the beam inducing the smallest optical switching is equal to *I*_0_, while the maximal intensity, causing the entire sample to optically switch (saturate) is equal to *I*_S_. The NA-dependent waist diameter of the saturating writing beam is equal to 2*w*_S_ (measured at 1/*e*^2^ of the peak intensity *I*_S_).

**Fig. 8:**
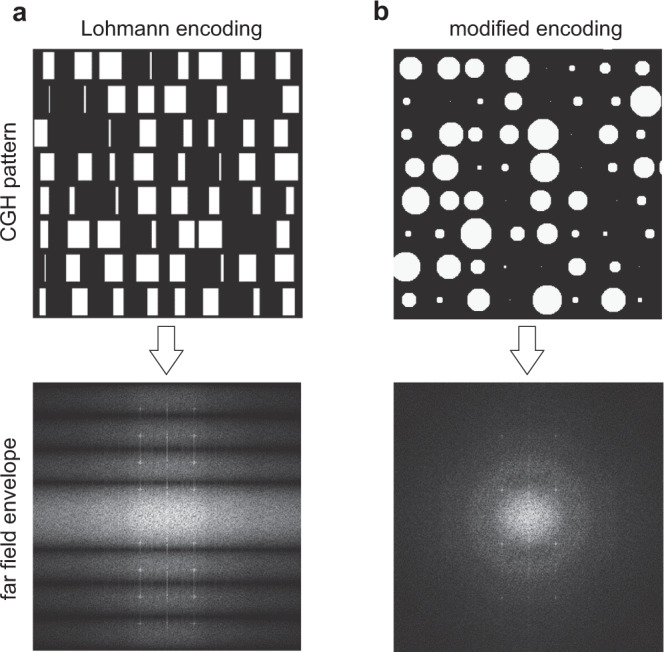
Complex encoding of CGH. Shaping of the far-field intensity envelope with modified Lohmann encoding comprising circular openings.

The rectangular openings in the Lohmann cells were replaced with circular ones, with the effect of symmetrical intensity envelope in far-field holographic playback field, attenuating higher-order terms (see Fig. 8). Since the CGH plane (*x*_1_,*y*_1_) and far-field playback plane (*x*_2_,*y*_2_) are in the Fourier relation, the attenuated reconstructed intensity field can be derived as:

4 $${{{{{\mathcal{F}}}}}}\left\{\left[h\left({x}_{1},{y}_{1}\right)\cdot {{{{{\rm{comb}}}}}}\left({x}_{1},{y}_{1}\right)\right]\otimes {{{{{\rm{circ}}}}}}\left({x}_{1},{y}_{1}\right)\right\}=\left[H\left({x}_{2},{y}_{2}\right)\otimes {{{{{\rm{comb}}}}}}\left({x}_{2},{y}_{2}\right)\right]\cdot {J}_{1}\left({x}_{2},{y}_{2}\right),$$

where *h* and *H* are the CGH data and the reconstructed image, respectively, *comb* function is the periodicity of the Lohmann cells, and *circ* function denotes the circular shape of the openings used for CGH encoding. The resultant Bessel function *J*_1_ is responsible for the symmetrical envelope, attenuating off-axis components in the playback signal.

### Point-by-point CGH computations for MEMS-driven opto-magnetic recording

Each pixel of a binary CGH must be written to a magneto-optical device in the order of nanoseconds in order not to stop the mechanical motion of the addressing MEMS mirror. Since conventional processors have difficulty meeting this requirement, we have developed FPGA-based (Field Programmable Gate Array) CGH processors^18^ that calculate a CGH point by the integration of contributions from all object points:

5 $${{{{{\rm{I}}}}}}\left({{{{{{\rm{x}}}}}}}_{h,}{y}_{h}\right)=\mathop{\sum }\limits_{j=1}^{N}{a}_{j}{{{{{\rm{cos}}}}}}\left(\frac{\pi \left({\left({x}_{h}-{x}_{j}\right)}^{2}+{\left({y}_{h}-{y}_{j}\right)}^{2}\right)}{{z}_{j}}\right),$$

where ${a}_{j}$ denotes the amplitude of an object point, $({{{{{{\rm{x}}}}}}}_{h,}{y}_{h})$ represents the coordinates of the computed CGH point, $({x}_{j},{y}_{j},{z}_{j})$ represents object points, $N$ denotes the total number of object points, and *I* is the output signal denoting the desired intensity of the pump beam exposing the CGH point (which in the binary case is equivalent to the desired transmittance of the CGH point). Those FPGA processors were designed to compute each CGH pixel in parallel, but the accumulation was done sequentially; thus, the calculation time is proportional to the number of object points. Therefore, it is not suitable for our purpose. For this opto-magnetic system, we have designed a new architecture of binary CGH processor. Equation (5) includes sequentially accumulation and the trigonometric function which consume hardware resources. In order to overcome this, we have derived a new CGH equation that can avoid these operations. In order to omit trigonometric functions, we first add the phase -π/2 to each object point, which does not affect reconstructed images. We can rewrite Eq. (5) as:

6 $${{{{{\rm{I}}}}}}\left({{{{{{\rm{x}}}}}}}_{h,}{y}_{h}\right)=\mathop{\sum }\limits_{j=1}^{N}{a}_{j}{{{{{\rm{sin}}}}}}\left(\frac{\pi \left({\left({x}_{h}-{x}_{j}\right)}^{2}+{\left({y}_{h}-{y}_{j}\right)}^{2}\right)}{{z}_{j}}\right),$$

In binary CGH calculation, the final result has only binary values, so we can approximate this equation as:

7 $${{{{{\rm{I}}}}}}\left({{{{{{\rm{x}}}}}}}_{h,}{y}_{h}\right)=\mathop{\sum }\limits_{j=1}^{N}{a}_{j}{{{{{\rm{T}}}}}}\left(\frac{\pi \left({\left({x}_{h}-{x}_{j}\right)}^{2}+{\left({y}_{h}-{y}_{j}\right)}^{2}\right)}{{z}_{j}}\right),$$

where the thresholding function $T(x)$ outputs ones if $T(x) \, < \, \pi$, otherwise zeros, which can be readily implemented into FPGA. In order to further omit the sequential accumulation, we simply expand the accumulation as:

8 $${{{{{\rm{I}}}}}}\left({{{{{{\rm{x}}}}}}}_{h,}{y}_{h}\right)={b}_{1}+{b}_{2}+\cdots+{b}_{N}.$$

where ${b}_{n}={a}_{n}{{{{{\rm{T}}}}}}\left(\frac{\pi (({{x}_{h}-{x}_{n}})^{2}+({{y}_{h}-{y}_{n}})^{2})}{{z}_{n}}\right)$. In order to reduce the usage of hardware resources, we set ${a}_{n}$ to 1. However, we can deal with arbitrary values of ${a}_{n}$ if we used an FPGA chip with larger resources. We can use the *popcount* technique (a.k.a Hamming weight) for Eq. (8), which can accelerate it to only log_2_(*N*) steps.

**Fig. 9:**
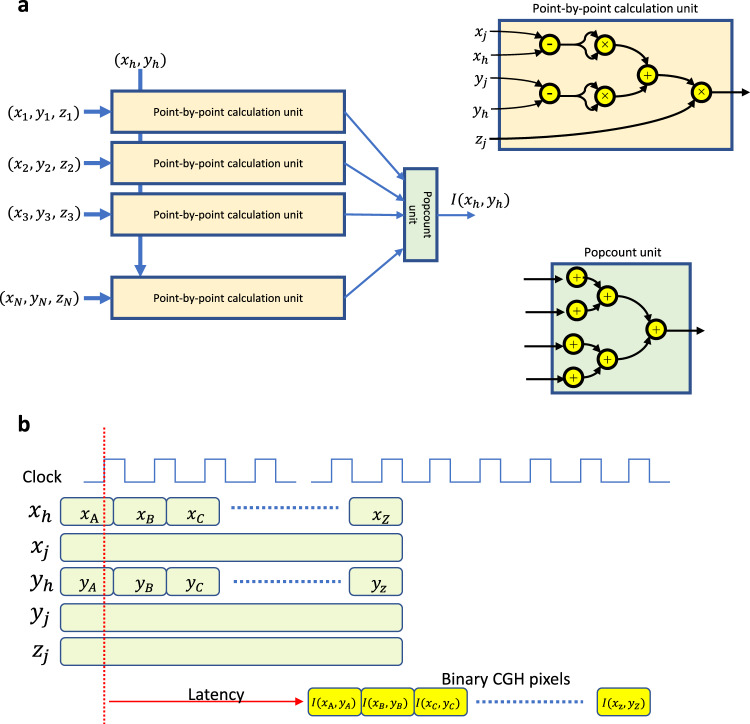
Serial computation of CGH. **a** A binary CGH processor for opto-magnetic recording with *popcount* unit for the accumulation of four binary bits. **b** Timing chart of the processor.

We have developed the binary CGH processor based on Eq. (8). Figure 9a shows a conceptual scheme of this circuit. The processor computes all object points in parallel and consists of a point-by-point calculation (PPC) unit and a *popcount* unit. A PPC unit calculates a CGH pixel at $({{{{{{\rm{x}}}}}}}_{h,}{y}_{h})$ from a single object point. In the case of $N$ object points, we prepare $N$ PPC units for each object point. Each PPC unit computes the same CGH coordinates, but computes different object points. In the current design, we can process the number of object points up to *N* = 2048 due to limited FPGA size, although in future ASIC^47^ implementations significantly higher *N* will be achieved with virtually no limitations. Figure 9b shows the timing chart of the processor. CGH pixels $I({{{{{{\rm{x}}}}}}}_{h,}{y}_{h})$ can be computed in only one clock (the order of nanosecond) because each unit was fully-pipelined. Before invoking the processor, we set $N$ object point data $({x}_{j},{y}_{j},{z}_{j})$ to the memory in FPGA. The processor calculates other binary CGH pixels by feeding other CGH coordinates $({{{{{{\rm{x}}}}}}}_{h,}{y}_{h})$ into the processor. We designed the PPC units with a 5-stage pipeline and the *popcount* unit with a 10-stage pipeline. Therefore, we need the latency of 15 clock cycles to obtain the first CGH pixel, but subsequent CGH pixels can be obtained within one clock cycle. It is worth noting that a single CGH pixel can be calculated by only one clock from *N* object points, while conventional CPUs and GPUs take *N* clock cycles; when *N* = 2048, our processor achieved over 2000 times acceleration as compared to the traditional approach. In addition, the FPGA chip does not need external memory for holding CGH pixels, therefore the processor can be readily applied to CGH calculations with a high spatial bandwidth product. Equations (6) and (7) will be further reduced in hardware resources by using a recurrence algorithm, allowing one to treat more object points^48,49^ in future iterations of the FPGA subsystem. Specifically, if the recurrence algorithm is included in the point-by-point FPGA calculation, one can reduce the circuit size to about one-fifth, allowing the treatment of about five times the number of object points with respect to the current implementation.

**Fig. 10:**
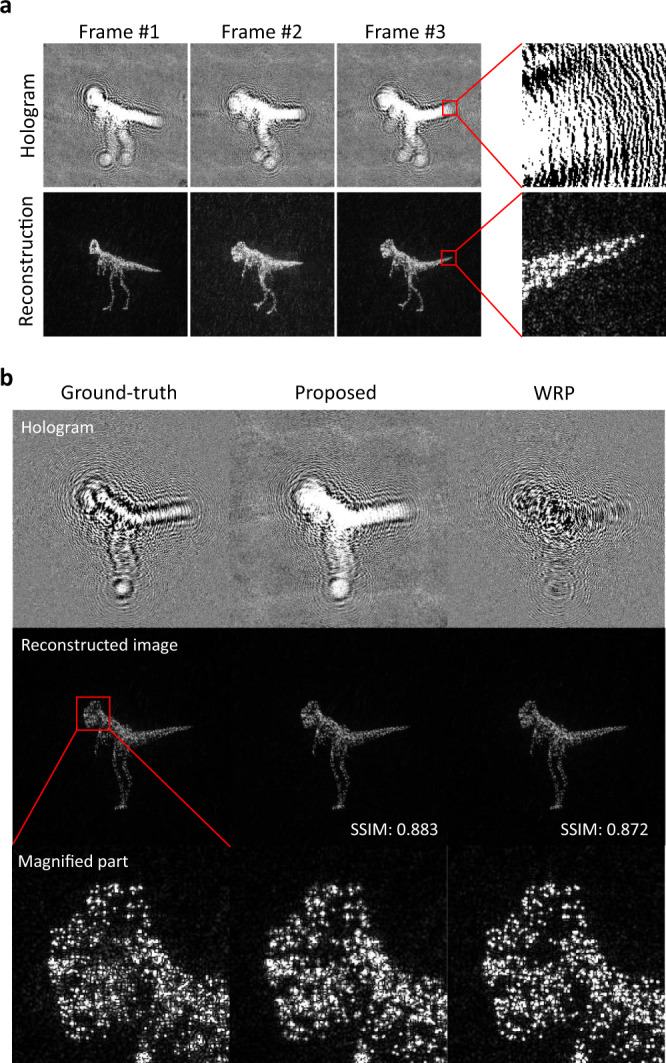
Quality of reconstructions from binary holograms. **a** Exemplary frames of a reconstructed movie from CGHs calculated by the FPGA processor. The top row shows the binary CGHs of the moving dinosaur, and the bottom row shows the corresponding reconstructed images. **b** CGHs computed with alternative algorithms and respective reconstructed images. Two frames of an animated dinosaur are computed by a direct computation of Eq. (5) (Ground-truth); the proposed method and wavefront recording plane (WRP) method. The same input object data and direct final amplitude binarization were used for fair comparison.

We have implemented the described binary CGH processor with 2048 PPC units and one *popcount* unit into a single FPGA chip of Virtex UltraScale+HBM FPGA (product part: xcvu35p-fsvh2104-3-e) made by Xilinx. The operation clock frequency was 125 MHz; thus, we could obtain one CGH pixel at 8 ns from 2048 object points. This can be scaled down to 1 ns by using modern FPGA chips with higher clock frequency up to 1 GHz without changing the proposed architecture, which was proven in FPGA simulations. The power consumption of the real FPGA CGH processor was about 14 W. The snapshot of a reconstructed movie from CGHs calculated by our processor is shown in Fig. 10a. The calculation conditions were that the CGH size of 1024 × 1024 pixels, the number of object points was 2048, the wavelength of 532 nm, and the distance of 0.3 m from the CGH. The input object was a 3-D model of a moving dinosaur. The used FPGA module has 1907 thousand FPGA logic cells, which is equivalent to 11 million ASIC logic cells. The available Intel e-ASIC N5X088 has 88 million ASIC logic cells, which potentially allows for about eight times more object points integrated at the same time in comparison to the implementation shown here. This combined with the above-mentioned optimization of the algorithm allows a roughly 40-fold improvement with commercially available technology. Therefore future implementations are open to feature a significantly larger number of object points, leading to higher quality holographic display of complex 3-D objects.

The influence of the used object point parallelization on the quality of the final CGH and its reconstruction is analyzed in Fig. 10b. The direct computation of the CGH according to Eq. (5) stands as the ground-truth reference. The proposed method is compared side-by-side with the alternative Wavefront Recording Plane (WRP) algorithm^50,51^, which is also based on the integration of the contributions from object points, followed by a wavefront propagation with scalar diffraction calculation (Angular Spectrum Method and Fresnel diffraction). In all three cases the binary amplitude was the resulting CGH data and holographic reconstruction was done with the Fourier transform. No significant error is noted, which is also supported by the shown structural similarity index (SSIM) values^52^. The SSIM values of the reference algorithm are lowered by the error caused by the final binarization of the computed fields, so as to adapt the results to the binary nature of the opto-magnetic writing medium. On the other hand, the proposed algorithm is binary in every step, therefore the quality and SSIM values for both algorithms are comparable within their standard deviations from frame to frame.

## Supplementary information

Supplementary Movie 1

Description of Additional Supplementary Files

## Data Availability

The authors declare that the data supporting the findings of this study are available within the article and its supplementary files.

## Code availability

The code is available from the corresponding authors upon reasonable request.

## References

[1] Shi L, Li B, Kim C, Kellnhofer P, Matusik W (2021). Towards real-time photorealistic 3D holography with deep neural networks. Nature.

[2] Peng Y., Choi S., Kim J. & Wetzstein G. Speckle-free holography with partially coherent light sources and camera-in-the-loop calibration, *Sci. Adv*. **7**,eabg5040 (2021). 10.1126/sciadv.abg5040 PMC8589315 34767449

[3] Blanche PA (2010). Holographic three-dimensional telepresence using large-area photorefractive polymer. Nature.

[4] Kobayashi Y, Abe J (2016). Real-time dynamic hologram of a 3D object with fast photochromic molecules. Adv. Opt. Mat..

[5] Li X (2015). Athermally photoreduced graphene oxides for three-dimensional holographic images. Nat. Commun..

[6] Blanche PA (2021). Holography, and the future of 3D display. Light.: Adv. Manufact..

[7] Vettese D (2010). Liquid crystal on silicon. Nat. Photon..

[8] Zhang Z, You Z, Chu D (2014). Fundamentals of phase-only liquid crystal on silicon (LCOS) devices. Light Sci. Appl..

[9] Tournilhac F, Blinov L, Simon J, Yablonsky SV (1992). Ferroelectric liquid crystals from achiral molecules. Nature.

[10] Kowalczyk A, Makowski M, Ducin I, Sypek M, Kolodziejczyk A (2018). Collective matrix of spatial light modulators for increased resolution in holographic image projection. Opt. Express.

[11] Sasaki H (2014). Large size three-dimensional video by electronic holography using multiple spatial light modulators. Sci. Rep..

[12] Blanche PA (2022). Towards a modular and scalable holographic display. Light Sci. Appl..

[13] Li J, Smithwick Q, Chu D (2022). Holobricks: modular coarse integral holographic displays. Light Sci. Appl..

[14] Shishido A (2010). Rewritable holograms based on azobenzene-containing liquid-crystalline polymers. Polym. J..

[15] Eralp M (2006). Submillisecond response of a photorefractive polymer under single nanosecond pulse exposure. Appl. Phys. Lett..

[16] Moon JS (2016). Sub-millisecond response time in a photorefractive composite operating under CW conditions. Sci. Rep..

[17] Yue Z, Xue G, Liu J, Wang Y, Gu M (2017). Nanometric holograms based on a topological insulator material. Nat. Commun..

[18] Sugie T (2018). High-performance parallel computing for next-generation holographic imaging. Nat. Electron..

[19] Horisaki (2018). Deep-learning-generated holography. Appl. Opt..

[20] Peng et al. Neural holography with camera-in-the-loop training. *ACM Trans. Graph.* **39**, 1–14 (2020).

[21] Yamamoto Y (2018). Large-scale electroholography by HORN-8 from a point-cloud model with 400,000 points. Opt. Express.

[22] Ostler TA (2012). Ultrafast heating as a sufficient stimulus for magnetization reversal in a ferrimagnet. Nat. Commun..

[23] Vahaplar K (2009). Ultrafast path for optical magnetization reversal via a strongly nonequilibrium state. Phys. Rev. Lett..

[24] Liu P, Sun X, Zhao Y, Li Z (2019). Ultrafast volume holographic recording with exposure reciprocity matching for TI/PMMAs application. Opt. Express.

[25] Finazzi M (2013). Phys. Rev. Lett..

[26] Wang Q (2016). Optically reconfigurable metasurfaces and photonic devices based on phase change materials. Nat. Photon..

[27] Xiao S, Davison I, Mertz J (2021). Scan multiplier unit for ultrafast laser scanning beyond the inertia limit. Optica.

[28] Davies CS (2020). Pathways for single-shot all-optical switching of magnetization in ferrimagnets. Phys. Rev. Appl..

[29] Hossack WJ, Theofanidou E, Crain J, Heggarty K, Birch M (2003). High-speed holographic optical tweezers using a ferroelectric liquid crystal microdisplay. Opt. Express.

[30] Ruffato G, Massari M, Romanato F (2016). Diffractive optics for combined spatial- and mode- division demultiplexing of optical vortices: design, fabrication and optical characterization. Sci. Rep..

[31] Zhu L, Wang J (2014). Arbitrary manipulation of spatial amplitude and phase using phase-only spatial light modulators. Sci. Rep..

[32] Ruffato G, Massari M, Romanato F (2019). Multiplication and division of the orbital angular momentum of light with diffractive transformation optics. Light Sci. Appl..

[33] Barnes TH (1992). Reconfigurable freespace optical interconnections with a phase-only liquid-crystal spatial light modulator. Appl. Opt..

[34] Roelens MA (2008). Dispersion trimming in a reconfigurable wavelength selective switch. J. Lightwave. Technol..

[35] Jana K (2021). Reconfigurable electronic circuits for magnetic fields controlled by structured light. Nat. Photon..

[36] Goodman, JW. Introduction to Fourier Optics, McGraw-Hill, New York (1968).

[37] Brown BR, Lohmann AW (1966). Complex spatial filtering with binary masks. Appl. Opt..

[38] Starobrat J (2021). Suppression of spurious image duplicates in Fourier holograms by pixel apodization of a spatial light modulator. Opt. Express.

[39] Stupakiewicz A, Szerenos A, Afanasiev D, Kirilyuk A, Kimel AV (2017). Ultrafast nonthermal photo-magnetic recording in a transparent medium. Nature.

[40] Avilés‑Félix L (2021). All‑optical spin switching probability in [Tb/Co] multilayers. Sci. Rep..

[41] Ignatyeva DO (2019). Plasmonic layer-selective all-optical switching of magnetization with nanometer resolution. Nat. Commun..

[42] Liu T-M (2015). Nanoscale confinement of all-optical magnetic switching in TbFeCo - competition with nanoscale heterogeneity. Nano Lett..

[43] Xiong J, Hsiang EL, He Z, Zhan T, Wu ST (2021). Augmented reality and virtual reality displays: emerging technologies and future perspectives. Light Sci. Appl..

[44] Wang Y (2021). Amplification of 1.08 GHz repetition rate femtosecond laser pulses to 97 W average power by a fiber amplifier with up to 1 GHz repetition rate. OSA Contin..

[45] Mezrich RS (1970). Magnetic holography. Appl. Opt..

[46] Makowski M (2022). Overlapping effect in dense all-optical, point-by-point recording of holographic patterns in the ferrimagnetic alloy. J. Magn. Magn. Mater..

[47] Young-Ho S, Yoon-Hyuk L, Dong-Wook K (2017). ASIC chipset design to generate block-based complex holographic video. Appl. Opt..

[48] Shimobaba T, Ito T (2001). An efficient computational method suitable for hardware of computer-generated hologram with phase computation by addition. Comput. Phys. Commun..

[49] Shimobaba T, Hishinuma S, Ito T (2002). Special-purpose computer for holography HORN-4 with recurrence algorithm. Comput. Phys. Commun..

[50] Shimobaba T, Masuda N, Ito T (2009). Simple and fast calculation algorithm for computer-generated hologram with wavefront recording plane. Opt. Lett..

[51] Tsang P, Cheung W-K, Poon T-C, Zhou C (2011). Holographic video at 40 frames per second for 4-million object points. Opt. Express.

[52] Wang Z (2004). Image quality assessment: from error visibility to structural similarity. IEEE Trans. Image Process..

[53] Starobrat J (2020). Photo-magnetic recording of randomized holographic diffraction patterns in a transparent medium. Opt. Lett..

