# Supplementary material for: Dynamic complex opto-magnetic holography
Source: Nat Commun. 2022 Nov 26;13:7286. doi: 10.1038/s41467-022-35023-9 (PMC9701213; doi:10.1038/s41467-022-35023-9)
Supplement: Supplementary file 2 — Description of Additional Supplementary Files [file 41467_2022_35023_MOESM2_ESM.pdf]

Title: Supplementary Movie 1.
Description: Exemplary dynamic holographic playback. The movie shows the dynamical sequence of opto-magnetic holographic reconstruction, demonstrating the counting between 0 and 9.
